# The mediation effects of nightmares and depression between insomnia and suicidal ideation in young adults

**DOI:** 10.1038/s41598-024-58774-5

**Published:** 2024-04-26

**Authors:** Zixuan Guo, Xiaoli Han, Tiantian Kong, Yan Wu, Yimin Kang, Yanlong Liu, Fan Wang

**Affiliations:** 1 Medical Neurobiology Lab, Inner Mongolia Medical University, Huhhot, 010110 China; 2 Beijing Hui-Long-Guan Hospital, Peking University, Beijing, 100096 China; 3 Clinical Nutrition Department, Friendship Hospital of Urumqi, Urumqi, 830049 China; 4 Xinjiang Key Laboratory of Neurological Disorder Research, The Second Affiliated Hospital of Xinjiang Medical University, Urumqi, 830063 China; 5 School of Mental Health, Wenzhou Medical University, Wenzhou, 325035 China

**Keywords:** Insomnia, Nightmare, Depression, Suicidal ideation, Mediation, Health care, Risk factors

## Abstract

Suicide is prevalent among young adults, and epidemiological studies indicate that insomnia, nightmares, and depression are significantly associated with a high incidence of suicidal ideation (SI). However, the causal relationship between these factors and SI remains unclear. Therefore, the purpose of this study was to examine the association between nightmares and depression and insomnia and SI in young adults, as well as to develop a mediation model to investigate the causal relationship between insomnia, nightmare, depression, and SI. We assessed insomnia, nightmares, depression, and SI in 546 young adults using the Insomnia Severity Scale (ISI), Disturbing Dream and Nightmare Severity Scale (DDNSI), Depression Study Scale (CESD-20), and Columbia-Suicide Severity Rating Scale (C-SSRS). Using the Bootstrap method, the mediation effects of nightmares and depression between insomnia and SI were calculated. The results demonstrated that nightmares and depression fully mediated the relationship between insomnia and SI, including the chain-mediation of insomnia and SI between nightmare and depression with an effect value of 0.02, 95% CI 0.01–0.04, and depression as a mediator between insomnia and SI with an effect value of 0.22, 95% CI 0.15–0.29. This study found that depression and nightmares may be risk and predictive factors between insomnia and SI, which implies that the assessment and treatment of depression and the simple or linked effect of nightmares play crucial roles in preventing SI in young adults.

## Introduction

Suicide is a devastating public health concern with profound personal and social implications in modern society^1^. As of 2019, more than 700,000 individuals died by suicide annually worldwide^1^, resulting in nearly $490 billion in medical and quality-of-life costs^2^. Despite ongoing research into various suicide prevention strategies, suicide rates are on the rise across the globe, and the World Health Organization has declared reducing suicide-related mortality a “global priority”^1^. Suicide is a complex phenomenon that is not yet completely comprehended, and suicidal ideation (SI) is the earliest stage and most significant risk factor^3,4^. SI is defined as a desire for death or, at least, an apathy toward life, which a specific suicide plan may accompany^5^. Therefore, it is essential to identify the factors that influence the emergence of SI.

Multiple factors may cause SI; however, certain sleep disorders (such as insomnia and nightmares) have a unique relationship with SI^6^. Insomnia is defined by the fifth edition of the American Diagnostic and Statistical Manual of Mental Disorders as a sleep disorder characterized by frequent difficulty falling asleep and/or difficulty maintaining sleep, resulting in inadequate sleep. It is one of the most common forms of sleep disorders in clinical practice^7,8^. Recent evidence identifies insomnia as a stable, independent predictor of suicide and a prevalent symptom among suicidal individuals^5,9^. A large adult sleep survey in the United States revealed a significant association between insomnia and SI^5,10^. In addition to insomnia, nightmares are a risk factor for suicide^11,12^. Nightmares are disturbing dreams during rapid eye movement (REM) sleep^5^. Frequent nightmares have been found to increase the risk of suicide by 1.5–3 times^13^. In a study examining the association between nightmares and suicide in adults, those who reported periodic nightmares had a 57% greater risk of suicide than those without nightmares, and those who reported frequent nightmares had a suicide risk of up to 107%^14^. In the past decade, nightmares received increasing attention as a potential mediator between insomnia and suicide. It has been reported that insomnia patients are more likely than non-insomnia patients to experience frequent nightmares, and nightmares may play a role in the relationship between insomnia and SI^15^. In addition, there is evidence that, although nightmares and insomnia symptoms are both associated with SI, they are not causally related^16^. A limited number of prospective studies have demonstrated that nightmares coincide with mental illness and may also serve as an early indicator of psychotic onset episodes. Chronic and recurrent nightmares are distressing to the individual and associated with varying degrees of depressive symptoms^17^. Insomnia and SI are also associated with depression^18,19^. Depression is a series of syndromes with low mood, decreased interest, and loss of pleasure as the core symptoms, which have become the focus of attention because of their high incidence, recurrence rate, and high risk of suicide^20^. A cross-sectional survey of 583 college students revealed that the relationship between insomnia and SI was fully mediated by depressive symptoms, suggesting that depression may be an additional important factor in developing insomnia and SI^16,21^.

In conclusion, the present study demonstrates that insomnia, nightmares, and depression are independent of each other that contribute to the manifestation of SI. However, it has not been definitively demonstrated that these factors are interconnected and causally associated with SI. Young adults are particularly vulnerable to the risk of suicide. Although there are notable variations in suicide rates based on factors such as age, gender, and geographical location, this demographic continues to be a primary focus for initiatives aimed at prevention and intervention ^22^. Therefore, it was hypothesized that nightmares and depression may mediate the relationship between insomnia and SI in young adults. This study aimed to design a mediation model to investigate the association between insomnia, nightmares, depression, and SI among young adults. The findings of this study can potentially contribute to the development of therapeutic interventions aimed at avoiding suicidal behaviors in this population.

## Materials and methods

### Data sources

The data utilized in this study were gathered from University of Arizona students aged 18–25 years, specifically during May 2020 and May 2021. These data were obtained as part of a Phase I survey to assess the impact of nocturnal sleep/wake effects on the risk of suicide, known as the Assessing Nocturnal Sleep/Wake Effects on Risk of Suicide (ANSWERS) project. Data is gathered through participation in undergraduate psychology courses as well as the distribution of flyers and emails. Prior to completing the survey, participants were required to provide informed consent through an electronic consent framework. The study was approved by the University of Arizona IRB (protocol # 2005675654). The relevant information about the ANSWERS can be accessed at: 10.25822/0vvb-6t89. The NSRR is supported by the National Institutes of Health, National Heart, Lung, and Blood Institute (R24 HL114473, 75N92019R002).

### Study methods

**Figure 1:**
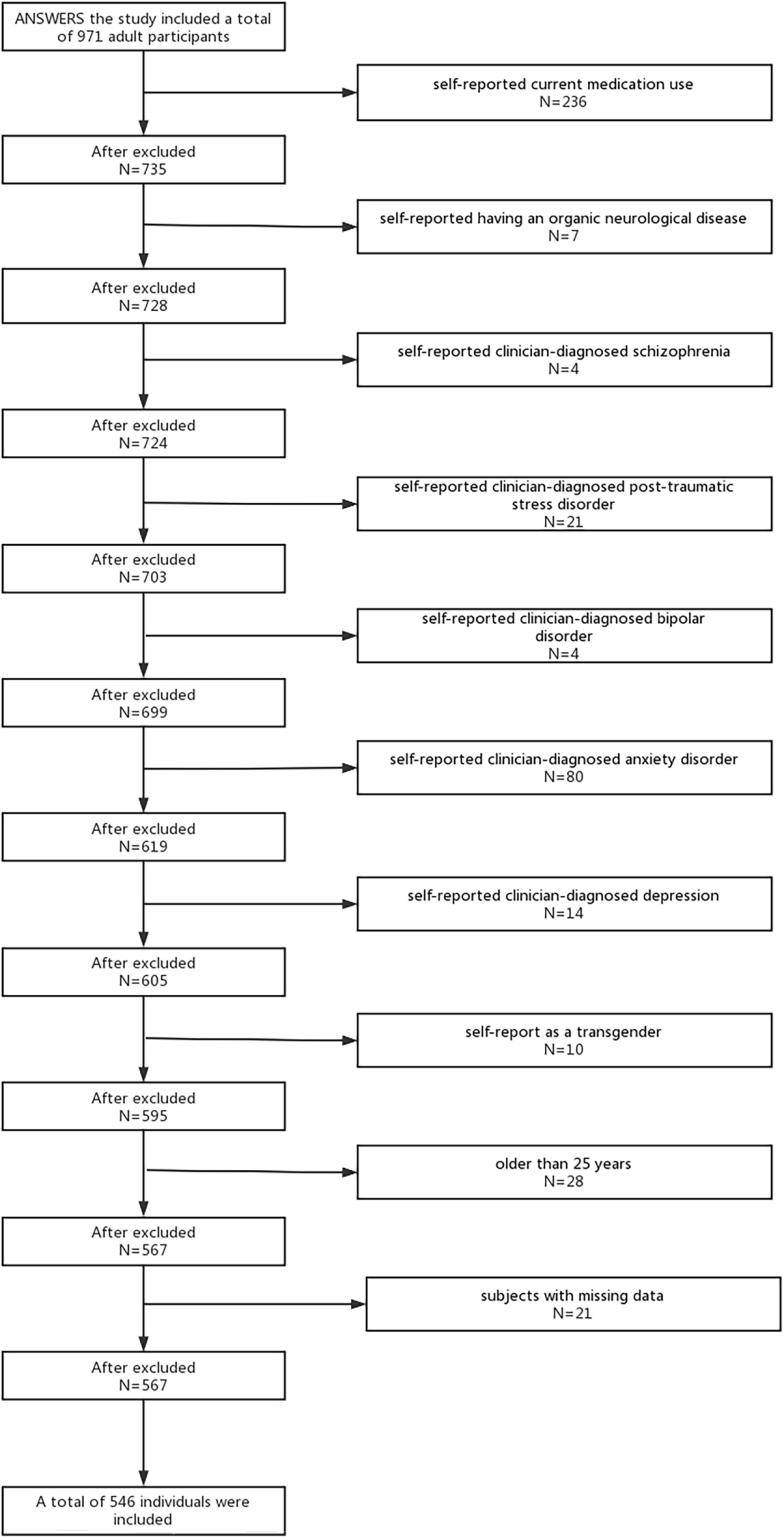
Flowchart of the population included in our final analysis.

In this study, Fig. 1 shows the collation and exclusion of the NSRR ANSWERS database. Our exclusion criteria included the following: self-reported current medication use, self-reported having an organic neurological disease, self-reported clinician-diagnosed schizophrenia, self-reported clinician-diagnosed post-traumatic stress disorder (PTSD), self-reported clinician-diagnosed bipolar disorder, self-reported clinician-diagnosed anxiety disorder, self-reported clinician-diagnosed depression, self-report as a transgender, older than 25 years, subjects with missing data. Our study ultimately included 546 participants. More than half of the 546 participants we included were non-Hispanic (66.7%), white (74.5%), and male (66%). The age ranged from 18 to 25 years, with an average age of 19.61 years ± 1.33 years. The vast majority (95.2%) had less than a high school education, and the majority (63.2%) reported an annual income of $25,000. The majority of participants (57.3%) revealed their drinking practices and intake of caffeine-containing items, while a smaller percentage reported a history of drug use (26.0%) and current smoking (14.8%).

Demographic Questionnaire: Sociodemographic variables included age, gender, sexual orientation, race, ethnicity, education level, and annual income. The survey included questions regarding behavioral attributes such as the consumption of alcohol, caffeinated products, cannabis, and current smoking habits.

Insomnia Severity Scale (ISI): This 7-item scale is a simple screening instrument for insomnia. The Likert scale was used to evaluate each item, with scores ranging from 0 to 4, with a maximum score of 28. The higher the score, the more pronounced the symptoms of insomnia, and when the score exceeds 14, clinically significant insomnia is identified. ISI exhibited excellent internal consistency alpha (α) = 0.84^23^. In this study, the Cronbach α and construct validity were 0.79 and 0.75, respectively.

Disturbing Dream and Nightmare Severity Index (DDNSI): This scale consisted of five questions that assessed self-reflective measures of the frequency and intensity of current nightmares. There are two sections on the frequency and number of nightmares per unit of time and the severity and intensity of nightmare problems. The scale also measures the incidence of nightmare-related awakenings. The score is obtained by measuring the number of nightmares per week, the number of nights with nightmares, the frequency of waking up with nightmares, the severity score, and the intensity score. The total score ranges from 0 to 37, and previous studies have indicated that a score greater than 10 indicates a nightmare disorder^24,25^. The scale is derived from the Nightmare Frequency questionnaire and revised with a Cronbach α of 0.80, indicating excellent internal consistency^26^. In this study, the Cronbach α and construct validity were 0.88 and 0.81, respectively.

Centre for Epidemiologic Studies Depression (CESD): The CESD is a 20-question self-report scale. Each item is scored between 0 and 3, with the total score ranging from 0 to 60. With definite depressed symptoms, the overall score exceeds 20. It is an efficient screening tool for detecting depressive symptoms, with α = 0.85 for the general population^27^. In this study, the Cronbach α and construct validity were 0.77 and 0.92, respectively.

Columbia-Suicide Severity Rating Scale (C-SSRS): The derivation of the C-SSRS was initially conducted by researchers from Columbia University, the University of Pennsylvania, and the University of Pittsburgh^3^. The U.S. Food and Drug Administration recommends the C-SSRS for clinical studies^28^, and the U.S. Centers for Disease Control and Prevention have adopted it to describe and categorize suicidal ideation and behavior^29^. This measure is used to evaluate the past 3 months and lifetime suicidal behavior and SI. The SI contained five items, three related to suicide conduct. All questions had a “0” for “no” and a “1” for “yes” response and the total of the five questions related to suicidal ideation was used to determine the severity of such ideation. In this study, the Cronbach α and construct validity were 0.75 and 0.77, respectively.

### Statistical analysis

The statistical analyses were performed using IBM SPSS Statistical System, version 22.0 (IBM Corporation in Armonk, New York, USA). The figures were generated with GraphPad 7 (GraphPad Software Company). This study used self-report measures to collect data, which may be susceptible to common methodological biases. To mitigate these biases, appropriate measures were taken during the data collection phase. These measures included ensuring participant anonymity and providing clear instructions to participants regarding the purpose of the study. The Harman Univariate test was used for statistical control before data analysis. Additionally, the items of all variables were subjected to analysis for non-rotating principal component factors. The findings indicate that 23 factors exhibited distinctive roots greater than 1. Furthermore, the extent of variation in the interpretation of the maximum factor was 28.18%, which is less than the cut-off value of 40%. As a result, it can be concluded that the data in this study does not exhibit any significant common method bias.

Initially, the Kolmogorov–Smirnov normality test and Levene’s test for homogeneity of variance were used for all continuous variables. Not all continuous variables had a normal distribution. Age was consistent with homogeneity of variance, using the one-way ANOVA test. Except for age, none of the continuous variables exhibited homogeneity of variance. Therefore, we employed the Mann–Whitney rank sum test to assess the observed differences. For all categorical variables, chi-square tests were used. The Mean ± standard deviation (SD) statistical measures were employed to describe continuous data, whereas frequency and percentage were utilized to depict categorical variables. Furthermore, Spearman’s correlation analysis examined the relationship between DDNSI, ISI, CESD, and C-SSRS scores. Additionally, we conducted a regression analysis between demographics and the outcome variables and selected the most appropriate control variables to reduce the confounding factors in the results. Finally, the mediation role between nightmares and depression in insomnia and SI was investigated using SPSS PROCESS v4.0. ISI was the independent variable, C-SSRS was the dependent variable, DDNSI was the mediator variable 1 (M1), CESD was the mediator variable 2 (M2), and the control variable produced through regression analysis was the covariate. All tests were two-sided, and the level of statistical significance was at *p* < 0.05.

### Ethics approval

All procedures followed were in accordance with the ethical standards of the responsible committee on human experimentation (University of Arizona) and with the Helsinki Declaration of 1975, as revised in 2000. Informed consent was obtained from all patients for being included in the study. The study was approved by the University of Arizona IRB (protocol # 2005675654).

### Informed consent

Data is gathered through participation in undergraduate psychology courses as well as the distribution of flyers and emails. Prior to completing the survey, participants were required to provide informed consent through an electronic consent framework.

## Results

### Demographics

**Figure 2:**
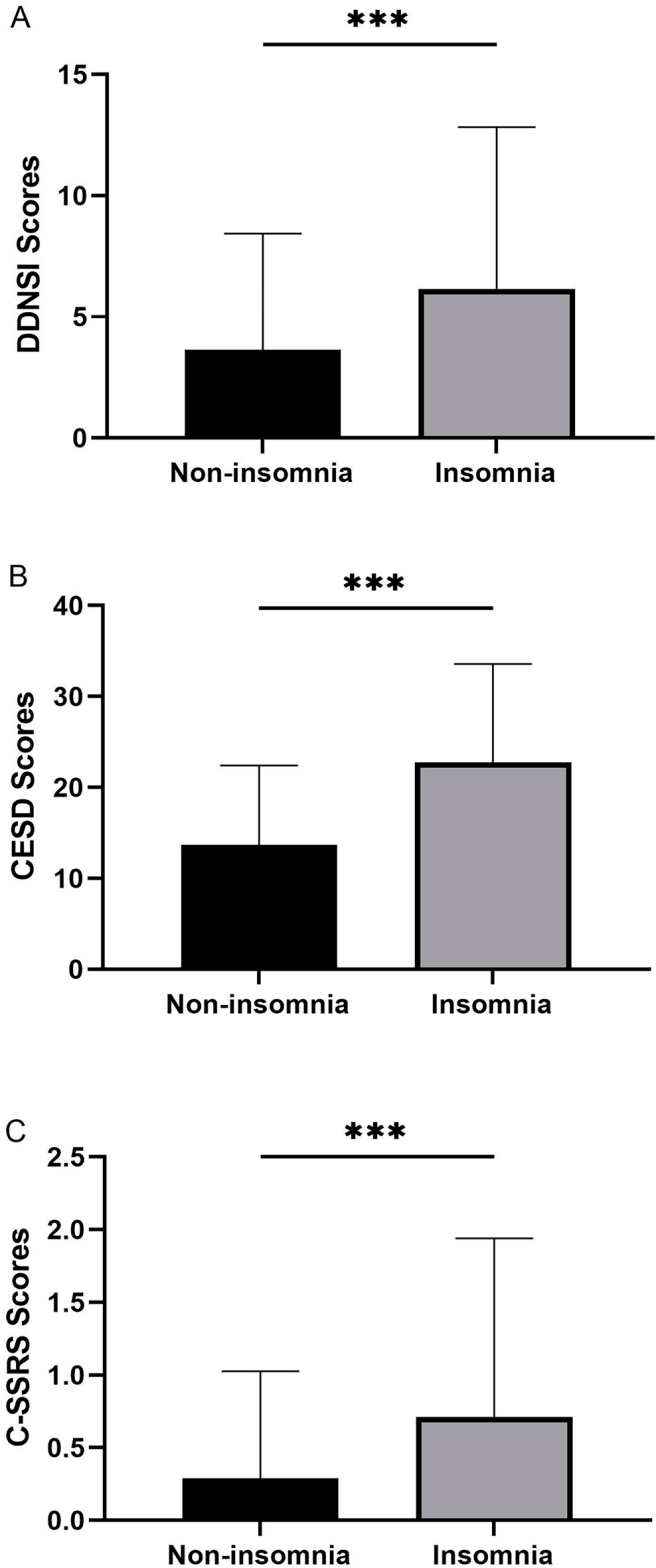
Differences between psychometric scales between insomnia and non-insomnia groups (n = 546). (**A**) Difference in DDNSI (Disturbing Dream and Nightmare Severity Index) total scores between insomnia and no insomnia group. (**B**) Difference in total CESD (Centre for Epidemiologic Studies Depression) scores between insomnia and no-insomnia groups. (**C**) Differences in C-SSRS (Columbia-Suicide Severity Rating Scale) scores between the insomnia and non-insomnia groups. ***p < 0.001.

Table 1 shows that the age of the insomnia group (19.75 ± 1.38 vs. 19.50 ± 1.27, *p* = 0. 030), the nightmare Index (6.15 ± 6.68 vs. 3.63 ± 4.80, *p* < 0.001), depression (22.76 ± 10.80 vs. 13.67 ± 8.76, *p* < 0.001), and SI (0.71 ± 1.23 vs. 0.29 ± 0.74, *p* < 0.001) were all higher than the non-insomnia group. The differences between the two groups are presented in Fig. 2. No statistically significant differences were found between the two groups regarding gender, race, education, income, alcohol drinking, and coffee consumption (all *p* > 0.05).

**Table 1:** Characteristics of participants with/without insomnia (n = 546).

Variables	Over all (n = 546)	Non-insomnia (n = 300)	Insomnia (n = 246)	F/χ^2^	*p*
Demographic questionnaire					
Age(years)	**19.61 ± 1.33**	**19.50 ± 1.27**	**19.75** **± 1.38**	**4.75**	0.030*
Gender (%)				0.13	0.715
Male	364 (66.7)	198 (36.3)	166 (30.4)		
Female	182 (33.3)	102 (18.7)	80 (14.7)		
Orientation (%)				1.41	0.235
Heterosexual	495 (90.7)	276 (50.5)	219 (40.1)		
Other	51 (9.3)	24 (4.4)	27 (4.9)		
Race (%)				8.12	0.150
White/Caucasian	407 (74.5)	232 (42.5)	175 (32.1)		
Black/African American	22 (4.0)	13 (2.4)	9 (1.6)		
Native American/Alaska Native	11 (2.0)	6 (1.1)	5 (0.9)		
Multiracial	28 (5.1)	9 (1.6)	19 (3.5)		
Native Hawaiian/Pacific Islander	1 (0.2)	1 (0.2)	0 (0.0)		
Asian American	77 (14.1)	39 (7.1)	38 (7.0)		
Ethnicity (%)				2.13	0.144
Non- Hispanic	364 (66.7)	208 (38.1)	156 (28.6)		
Hispanic	182 (33.3)	92 (16.8)	90 (16.5)		
Education (%)				0.27	0.604
College or more	26 (4.8)	13 (2.4)	13 (2.4)		
High school or less	520 (95.2)	287 (52.6)	233 (42.7)		
Income (%)				4.93	0.177
< 25,000 dollars	345 (63.2)	185 (33.9)	160 (29.3)		
25,000–200000dollars	44 (8.1)	25 (4.6)	19 (3.5)		
> 200000dollars	13 (2.4)	11 (2.0)	2 (0.4)		
No report	144 (26.4)	79 (14.5)	65 (11.9)		
Behavior characteristics					
Drink alcohol (%)				3.04	0.081
No	233 (42.7)	118 (21.6)	115 (21.1)		
Yes	313 (57.3)	182 (33.3)	131 (24.0)		
Drink caffeinated products (%)				0.12	0.727
No	56 (10.3)	32 (5.9)	24 (4.4)		
Yes	490 (89.7)	268 (49.1)	222 (40.7)		
Use marijuana/cannabis (%)				0.16	0.692
No	404 (74.0)	224 (41.0)	180 (33.0)		
Yes	142 (26.0)	76 (13.9)	66 (12.1)		
Current smoking (%)				1.78	0.183
No	465 (85.2)	261 (47.8)	204 (37.4)		
Yes	81 (14.8)	39 (7.1)	42 (7.7)		
DDNSI scores	**4.77 ± 5.85**	**3.63 ± 4.80**	**6.15 ± 6.68**	**4.53**	< 0.001***
CESD scores	**17.77 ± 10.73**	**13.67 ± 8.76**	**22.76 ± 10.80**	**9.75**	< 0.001***
C-SSRS scores	**0.48 ± 1.01**	**0.29 ± 0.74**	**0.71 ± 1.23**	**4.39**	< 0.001***

DDNSI, Disturbing Dream and Nightmare Severity Index; CESD, Centre for Epidemiologic Studies Depression; C-SSRS, Columbia-Suicide Severity Rating Scale.

Variables using percentage are reported as a chi-square test between with/without insomnia. Age was tested using a one-way variance test between two groups, and all other data using the Man-Whitney rank sum test. All data were reported as Mean ± SD. *p <0 .05, ** *p* < 0.01, ***p <0 .001.

Significance values are bold.

### Correlation analysis

Table 2 displays the correlations between insomnia, nightmares, SI, and depression. Insomnia was positively associated with nightmares, depression, and SI (*p* < 0.01). Nightmares were positively associated with depression and SI (*p* < 0.01). Depression scale scores positively correlated with SI (*p* < 0.01).

**Table 2:** Pearson correlations between the individual variables.

Variables	ISI scores	DDNSI scores	CESD scores	C-SSRS scores
ISI scores	1.00			
DDNSI scores	0.27**	1.00		
CESD scores	0.50**	0.29**	1.00	
C-SSRS scores	0.24**	0.15**	0.49**	1.00

ISI, Insomnia Severity Index; DDNSI, Disturbing Dream and Nightmare Severity Index; CESD, Centre for Epidemiologic Studies Depression; C-SSRS, Columbia-Suicide Severity Rating Scale.

The association between the variables was calculated using the double correlation method. **p <0 .01.

### Regression analysis

Research shows that age^30,31^, gender^32,33^, sexual orientation^34–36^, education^37,38^, income^39,40^, alcohol consumption^41,42^, and cannabis use^43–45^ can affect suicide. To rule out the influence of confounding variables, we used the SI as the dependent variable and conducted a linear regression analysis on the variables above. As shown in Table 3, only sexual orientation was statistically significant (*p* < 0.001), so we accounted for it as a covariable in the subsequent analysis.

**Table 3:** Regression relationships between C-SSRS Scores and predictor variables.

		R^2^	F	B	β	t	*p*
C− SSRS scores	Age	0.04	3.15	− 0.05	− 0.04	− 0.94	.350
	Gender			− 0.15	− 0.05	− 1.14	.255
	Orientation			0.84	0.17	3.91	< .001***
	Education			0.12	0.02	0.40	.688
	Income			− 0.06	− 0.05	− 1.21	.227
	Drink alcohol			− 0.04	− 0.02	− 0.32	.753
	Use marijuana/cannabis			0.14	0.04	0.91	.362

R^2^, coefficient of determination; B, unstandardized regression coefficient; β, standardized regression coefficient; C-SSRS, Columbia-Suicide Severity Rating Scale.

Stepwise multiple regression analysis was used to predict the correlation between variables. *p* < 0.05 was considered significant. ***p <0 .001.

### Mediation analysis

**Figure 3:**
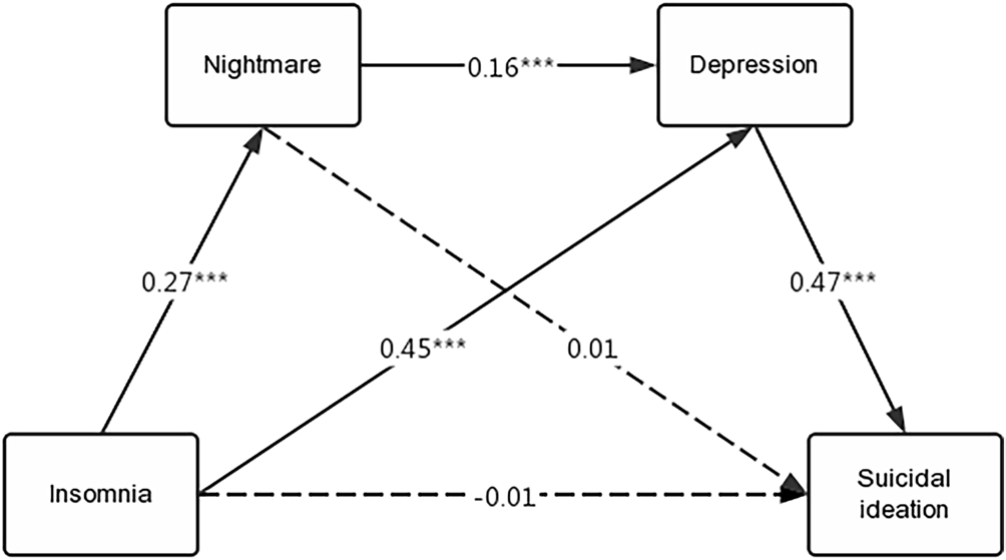
Mediation model of nightmares and depression between insomnia and suicidal ideation Figure (The solid lines are the paths with significant path coefficients, and the dashed lines are the non-significant paths).

Correlation analysis revealed a pairwise correlation between the four study variables; therefore, the mediation model could be used to investigate the mediation effect between the four variables. Using ISI scores as the independent variable, C-SSRS scores as the dependent variable, DDNSI and CESD scores as M1 and M2 mediation variables, and sexual orientation scores as control variables. Table 4 and Fig. 3 depicts multiple hierarchical regression analysis, which was performed using the non-parametric percentile-guided method PROCESS v.4.0 model 6 proposed by Hayes. Insomnia had a significant positive effect on nightmares (B = 0.27, *p* < 0.001) and depression (B = 0.45, *p* < 0.001), and depression had a significant positive effect on SI (B = 0.47, *p* < 0.001).

**Table 4:** Regression analysis of the variable relationship in the mediation model.

Regression equation		Overall fit index			Regression coefficient		
Outcome variables	Predictor variables	R	R^2^	F	B	t	95%CI
Model 1 (DDNSI)	Orientation	0.28	0.08	22.24	0.09	1.29	(− 0.05, 0.23)
	ISI				0.27	6.43***	(0.18, 0.35)
	DDNSI				–	–	–
	CESD				–	–	–
Model 2 (CESD)	Orientation	0.53	0.28	70.19	0.11	1.73	(− 0.01, 0.23)
	ISI				0.45	11.96***	(0.38, 0.53)
	DDNSI				0.16	4.21***	(0.09, 0.23)
	CESD				–	–	–
Model 3 (C− SSRS)	Orientation	0.50	0.25	45.51	0.21	3.36**	(0.09, 0.34)
	ISI				− 0.01	− 0.25	(− 0.10, 0.07)
	DDNSI				0.01	0.24	(− 0.07, 0.09)
	CESD				0.47	10.84***	(0.39, 0.56)
Model 4 (C− SSRS)	Orientation	0.29	0.08	24.44	0.27	3.89***	(0.13, 0.41)
	ISI				0.23	5.51***	(0.15, 0.31)
	DDNSI				–	–	–
	CESD				–	–	–

R^2^, coefficient of determination; B, unstandardized regression coefficient; ISI, Insomnia Severity Index; DDNSI, Disturbing Dream and Nightmare Severity Index; CESD, Centre for Epidemiologic Studies Depression; C-SSRS, Columbia-Suicide Severity Rating Scale.

All continuous variables in the model were standardized before substitution into the equation. *p < 0.05, **p < 0.01, ***p < 0.001.

The mediation effect between insomnia and SI was examined using the Bootstrap method, which was repeated 5000 times to determine the significance of the mediation effect, with a 95% confidence interval (CI). The relationship between insomnia and SI was completely mediated by nightmares and depression, with a total effect value of 0.24, as shown in Table 5. Among these indirect effects are two distinct pathways. First, depression as a mediator had a 0.22 effect size on insomnia and SI. Second, the relation between nightmares and depression mediated the association between insomnia and SI with an effect size of 0.02. The Bootstrap 95% CI did not contain 0 for both indirect effects, and both reached significant levels. This shows that depression can have a role in insomnia and SI alone and mediate insomnia and SI through linking effects with nightmares.

**Table 5:** Mediation analysis.

	Effect	BootSE	BootLLCI	BootULCI	Relative proportion (%)
Total indirect effect	0.24	0.04	0.17	0.31	
ISI → DDNSI → C-SSRS	0.00	0.01	− 0.02	0.03	–
ISI → CESD → C-SSRS	0.22	0.03	0.15	0.29	91.7
ISI → DDNSI → CESD → C-SSRS	0.02	0.01	0.01	0.04	8.3

ISI, Insomnia Severity Index; DDNSI, Disturbing Dream and Nightmare Severity Index; CESD, Centre for Epidemiologic Studies Depression; C-SSRS, Columbia-Suicide Severity Rating Scale; SE, standard error; LLCI, lower limit of confidence interval; ULCI, upper limit of confidence interval.

## Discussion

To the best of our knowledge, this is the first study to examine the mediation association between nightmares and depression in insomnia and SI in a general youth population. This mediation model has not been verified in previous studies. The findings of this study confirm that insomnia causes SI through nightmares and depression as mediators. This effect of mediation includes two pathways. We found for the first time an 8.3% effect proportion for a linkage mediation effect of nightmares and depression between insomnia and SI. Secondly, we observed that the depression’s sole mediator effect was the largest at 91.7%. These findings provide a deeper understanding of the risk factors and intrinsic mechanisms underlying insomnia-mediated SI in youth, enabling us to prevent suicidal behavior more effectively.

Our primary findings suggest that nightmares and depression play a linkage role between insomnia and SI, although relatively weak, but nonetheless significant. The chain mediation effect of nightmares and depression may be due to abnormal hypothalamic–pituitary–adrenal axis (HPAA) activity from a biological perspective. Both animal and human studies indicate that insomnia causes HPAA hyperactivity^46^ and elevated levels of adrenocorticotropic hormone (ACTH) secreted by the pituitary gland. ACTH is an intraneural secretion that corresponds to the sympathetic nervous system and hyperarousal states^47^. Increasing activity in the HPAA axis promotes the fragmentation of REM sleep^47,48^, and nightmares may be a consequence of REM sleep fragmentation^16^. Insomnia increased the likelihood of having nightmares, which were substantially linked to an increase in depression^49^. Depression is characterized by pervasive disruption of the brain’s functional networks^50,51^. According to magnetic resonance imaging findings, orbital frontal cortex (OFC) and anterior cingulate cortex (ACC) in the prefrontal cortex (PFC), putamen and caudate in the hippocampus and amygdala^52^, and frontal-ACC-striatal circuit defects may underlie the biological mechanism of depression. Decision-making, and emotional regulation could be SI’s vulnerability factors^53^. The OFC is a subregion of the PFC that is a key region for emotion and impulse regulation, including the evaluation of decision actions and stimuli^54^, and its reduced volume and thickness may result in impaired emotion regulation, resulting in poor decision-making and impulsivity^55,56^. As a connection between the prefrontal cortex and the limbic striatal system, the ACC is associated with regulating impulsive behavior^57^. Despite the fact that not all SI is impulsive, impulsivity may be an indicator of suicide risk in patients with mental disorders^58,59^. As a component of the striatum, the putamen is frequently associated with motor skills and is involved in the process of reward^60,61^. In a depressed state, abnormalities in the putamen may result in decisions based on imminent rewards, thereby promoting suicidal impulses.

According to suicide research, suicide risk factors are more complex. In addition to biological factors, the interpersonal-psychological theory of suicide is one of the most prominent theories regarding suicidal behavior^62^. It is assumed that a frustrated sense of belonging and a burdensome perception contribute to SI^63^. A recent qualitative study showed that individuals with chronic insomnia accumulated negative effects during work and social activities, which may lead them to perceive themselves as a burden^27^. This negative-oriented perception (especially before bedtime) fosters negative dream content and thereby increases the likelihood of having nightmares, which is regarded as a key nightmare trigger^64^. Long-lasting nightmares can cause loss of control, depression, and even despair^27^. In a recent online cross-sectional survey, prolonged periods of negative affect played an important role in the association between nightmares and suicidal behavior^17,65–67^. Thus, we propose that insomnia increases the frequency of nightmares by increasing feelings of loneliness and frustrated belonging, resulting in negatively mediated SI.

Our secondary findings indicate that depression is a significant single mediator of insomnia and SI. Increasing evidence suggests that insomnia may increase the inflammatory cytokine interleukin-6 (IL-6) expression by enhancing nuclear factor-kappa B (NF-kB) activation and β-Adrenergic signaling^68,69^. A significant correlation exists between elevated inflammatory markers caused by insomnia and the subsequent development of depressive symptoms^70^. The depletion of peripheral tryptophan, the precursor to serotonin (5-HT), has been linked to IL-6-induced short-term mood alterations resembling depression, according to studies^71,72^. According to the monoamine deficiency theory, 5-HT deficiency in the central nervous system is the underlying pathophysiological cause of depression^73^. 5-HT is a monoamine neurotransmitter that is broadly distributed in the brain and is closely linked with the regulation of information processing and emotional behavior^74^. Insomnia also causes chronic changes in the sensitivity and density of the presynaptic 5-HT transporter and postsynaptic 5-HT receptors, resulting in a decrease in 5-HT levels in the brain^75,76^. Previous research has linked insufficient or impaired serotonin activity to suicidal behavior, and the association of low serotonin with depression, impulsivity, and aggression may explain this relationship^77,78^.

Lastly, the mediation effect proportion analysis revealed a statistically significant difference between the two paths’ effect proportions. Depression had the highest proportion (91.7%), and the effect of nightmares as a mediator between depression and nightmares was comparatively small (8.3%). This may be determined by the disease’s prevalence. According to numerous epidemiological studies, depression is the most common psychiatric disorder co-occurring with insomnia, afflicting 40% of insomnia patients^79^. Comorbidity rates of nightmares and depression were higher only in patients with PTSD and borderline personality disorder^80,81^, indicating that the related mediation of nightmares and depression was relatively low in our study’s population. Although lower than the path of depression, it is still a developmental trend, suggesting that we should pay attention to the relationship between nightmares and depression.

There are a number of limitations to this investigation. First, our current study focused on young adults between the ages of 18 and 25, which may limit the study’s applicability to other age groups. However, because the risk of suicidal behavior between these ages is so high^82^, we chose to concentrate on this population. Second, we did not exclude marijuana smokers from this study because the frequency of use rather than the exact number reported in the database makes it impossible to determine the exact amount. In addition, the prevalence of cannabis use has increased since 1980 and varies geographically^83^, so we have no control over cannabis use. Studies have shown that race and cultural issues can influence suicidal ideation and behavioral risk among emerging adults and college students^82^; however, we did not control the race of the included group, which may have affected our findings.

## Conclusion

This study is the first to demonstrate that nightmares and depression together mediated the relationship between insomnia and SI. In addition to playing a separate function in insomnia and SI, depression may also have a mediating effect with nightmares. Assessment and treatment of nightmares and depressed mood may have important implications for preventing suicidal behavior in young adults. Health professionals should take appropriate preventive measures.

## Data Availability

The relevant information about the ANSWERS can be accessed at: 10.25822/0vvb-6t89. The datasets generated during and/or analysed during the current study are available from the corresponding author on reasonable request.

## Abbreviations

SI: Suicidal ideation
REM: Rapid eye movement
NSRR: National sleep research resource
ANSWERS: Assessing nocturnal sleep/wake effects on risk of suicide
PTSD: Post-traumatic stress disorder
ISI: Insomnia severity index
DDNSI: Disturbing dream and nightmare severity index
CESD: Center for Epidemiologic Studies Depression
C-SSRS: Columbia-suicide severity rating scale
α: Alpha
SD: Standard deviation
NF-kB: Nuclear factor-kappa B
IL-6: Interleukin-6
5-HT: 5-Hydroxytryptamine
PFC: Prefrontal cortex
OFC: Orbital frontal cortex
ACC: Anterior cingulate cortex
HPAA: Hypothalamic–pituitary–adrenal axis
ACTH: Adrenocorticotropic hormone
ANOVA: One-way analysis of variance
R^2^: Coefficient of determination
B: Unstandardized regression coefficient
β: Standardized regression coefficient
CI: Confidence interval
SE: Standard error
LLCI: Lower limit of confidence interval
ULCI: Upper limit of confidence interval
